# Association of Complement Receptor 2 Gene Polymorphisms with Susceptibility to Osteonecrosis of the Femoral Head in Systemic Lupus Erythematosus

**DOI:** 10.1155/2016/9208035

**Published:** 2016-06-30

**Authors:** Tae-Ho Kim, Sang-Cheol Bae, Sang-Han Lee, Shin-Yoon Kim, Seung-Hoon Baek

**Affiliations:** ^1^Biomedical Research Institute, Kyungpook National University Hospital, 135 Dongdeok-ro, Jung-gu, Daegu 700-721, Republic of Korea; ^2^Skeletal Diseases Genome Research Center, Kyungpook National University, 135 Dongdeok-ro, Jung-gu, Daegu 700-721, Republic of Korea; ^3^Department of Rheumatology, Hanyang University Hospital for Rheumatic Diseases, 17 Haengdang-Dong, Seongdong-Gu, Seoul 133-792, Republic of Korea; ^4^Department of Food Science & Biotechnology, Kyungpook National University, 80 Daehak-ro, Buk-gu, Daegu 41566, Republic of Korea; ^5^Department of Orthopedic Surgery, Graduate School of Medicine, Kyungpook National University, 130 Dongdeok-ro, Jung-gu, Daegu 700-721, Republic of Korea

## Abstract

Osteonecrosis of the femoral head (ONFH) is a complex and multifactorial disease that is influenced by a number of genetic factors in addition to environmental factors. Some autoimmune disorders, including systemic lupus erythematosus (SLE), rheumatoid arthritis (RA), and inflammatory bowel disease (IBD), are associated with the development of ONFH. Complement receptor type 2 (*CR2*) is membrane glycoprotein which binds C3 degradation products generated during complement activation.* CR2* has many important functions in normal immunity and is assumed to play a role in the development of autoimmune disease. We investigated whether* CR2* gene polymorphisms are associated with risk of ONFH in SLE patients. Eight polymorphisms in the* CR2* gene were genotyped using TaqMan*™* assays in 150 SLE patients and 50 ONFH in SLE patients (SLE_ONFH). The association analysis of genotyped SNPs and haplotypes was performed with ONFH. It was found that three SNPs, rs3813946 in 5′-UTR (untranslated region), rs311306 in intron 1, and rs17615 in exon 10 (nonsynonymous SNP; G/A, Ser639Asn) of the* CR2* gene, were associated with an increased risk of ONFH under recessive model (*P* values; 0.004~0.016). Haplotypes were also associated with an increased risk (OR; 3.73~) of ONFH in SLE patients. These findings may provide evidences that* CR2* contributes to human ONFH susceptibility in Korean SLE patients.

## 1. Introduction

Osteonecrosis of the femoral head (ONFH) is a complex and multifactorial disease which can be affected by combined genetic factors with relatively small effect in addition to environmental factors [1]. A variety of conditions, such as use of corticosteroids, alcohol abuse, and rheumatic diseases were reported as risk factors for secondary ONFH. Among autoimmune diseases, systemic lupus erythematosus (SLE) has shown higher incidence of ONFH ranging from 5 to 30%, than that of general population and ONFH, in turn, results in significant morbidity [2]. Although corticosteroid use has been reported as a significant predictive factor for developing ONFH in patients with SLE [3], there are also reports of patients with SLE complicated by ONFH, who have not taken corticosteroid [4, 5]. This implicates possible role of the disease progression itself or underlying genetics. Although some studies reported that immunologic factors including interleukins and tumor necrosis factors might develop ONFH [6, 7], most genetic studies have focused on gene polymorphisms affecting the coagulation and fibrinolytic systems [8, 9]. Moreover, few genetic studies were performed to reveal their roles in the development of SLE_ONFH [10].

Human complement receptor 2 (*CR2*) is encoded by a single gene containing 20 exons, which is located at chromosome 1q32.2 [11] and is expressed on mature B or follicular dendritic cells (FDCs) [12].* CR2* was known to bind C3 degradation products generated during the process of complement activation [13] and some studies suggested that* CR2* might play an important role in immunity [14]. Therefore, given the pleiotropic effects of complement receptor, we investigated whether polymorphisms of the* CR2* gene are associated with the susceptibility of SLE_ONFH.

## 2. Material and Methods

### 2.1. Patients and DNA

Blood samples and medical records were obtained from 150 SLE patients (13 males, 137 females; mean age, 31.37 ± 9.97) and 50 SLE_ONFH patients (4 males, 46 females; mean age, 31.28 ± 9.04). The SLE and SLE_ONFH patients were consecutively recruited from the Hanyang University Hospital for Rheumatic Diseases (Seoul, Korea). All SLE patients met the American College of Rheumatology (ACR) criteria for the classification of SLE [15]. Genomic DNA was isolated from the peripheral blood of each participant using a FlexiGene DNA Kit (QIAGEN, Valencia, CA). The current study was approved by the Institutional Review Board, and all participants in this study provided their informed consent.

### 2.2. Genotyping

Eight single nucleotide polymorphism (SNP) sites in the CR2 gene were selected based on locations, potential relevance to disease, and published data [11, 16, 17]. The genotype was determined using a TaqMan fluorogenic 5′-nuclease assay with predesigned or custom TaqMan primer/probe sets (Applied Biosystems, Foster City, CA). For genotyping of polymorphic sites, amplification primers and probes were designed for TaqMan assays (Applied Biosystems, Foster City, CA). The primer and probe sequences are indicated in Table 1. We designed both the PCR primers and the minor groove binder (MGB) TaqMan probes using Primer Express (Applied Biosystems). All reactions were performed following the manufacturer's protocol. Details regarding the PCR reaction and TaqMan assay have been described previously [9]. The fluorescence data files from each plate were collected and analyzed using automated allele-calling software (SDS 2.2, Applied Biosystems).

### 2.3. Statistical Analyses

The threshold of Hardy-Weinberg equilibrium (HWE) *P* value is set at >0.05. Allelic or genotype association tests in the case-control were calculated using *χ* ^2^ test or Fisher's exact test. Odds ratios (ORs) and corresponding 95% confidence intervals (CIs) for case-control data were also calculated. Genotypes were given codes of 0, 1, and 2; 0, 1, and 1; and 0, 0, and 1 in the codominant, dominant, and recessive models, respectively. The strength of linkage disequilibrium (LD) among the pairs of SNPs was evaluated using Haploview 4.2 software (http://www.broad.mit.edu/mpg/haploview/). Haploview software was also used to calculate haplotype structures and their frequencies within LD blocks. Haplotypes with frequencies < 5% were excluded from the following analysis. Continuous variables were compared by Student's *t*-test or ANOVA. All analyses were two-tailed, and *P* values <0.05 were considered to be statistically significant.

## 3. Results

### 3.1. Genetic Association of CR2 SNPs with SLE_ONFH Susceptibility

**Figure 1:**
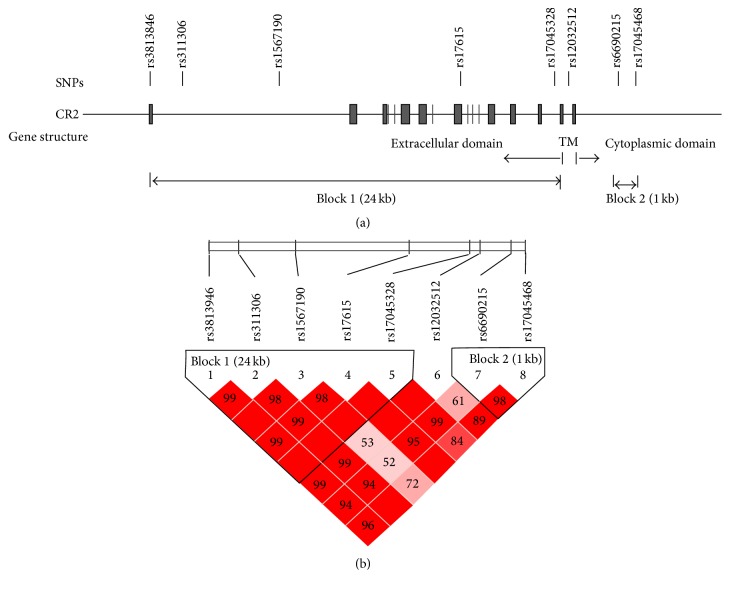
CR2 SNP locations and haplotype blocks. (a) The CR2 gene consists of 20 exons. Eight SNPs in the 5′ UTR, exon 10, and several intron regions were genotyped. (b) *D*′ and *r* ^2^ of each SNP pair are shown. Two haplotype blocks were constructed based on the strength of LD among SNP pairs. The first 5 SNPs formed 24 kb block 1 and next SNPs formed block 2 (see Table 5).

To determine whether* CR2* gene polymorphisms might contribute to the susceptibility of ONFH development in SLE patients of Korea (SLE_ONFH), the sample of 150 SLE and 50 SLE_ONFH Korean patients was genotyped using eight SNPs spanning a 39 kb region of the* CR2* gene from 0.6 kb upstream to 2.8 kb downstream of the gene (Figure 1). We selected 8 informative SNPs that included 1 regulatory SNP (rs3813946 in 5′-UTR; T/C), 1 exonic SNP (rs17615 in exon 10; G/A, Ser639Asn), and 6 haplotype-tagging intronic SNPs that tagged the two haplotype blocks (Figure 1(b)). The resulting SNP data including location, amino acid substitution, genotype, MAF, and HWE of all analyzed polymorphisms are demonstrated in Table 2.  Table 3 shows a comparison of genotype frequencies between case-control groups. When genotype distributions between the SLE (control) and SLE_ONFH (case) groups were compared, the SNPs rs3813946 in 5′-UTR (untranslated region), rs311306 in intron 1, and rs17615 in exon 10 (nonsynonymous SNP; G/A, Ser639Asn) of the* CR2* gene, located in block 1, demonstrated the evidence for association with risk of ONFH under recessive model (*P* values; 0.004~0.016). None of block 2 SNPs showed evidence for association (Table 3).

### 3.2. Association of CR2 SNP Haplotypes with SLE_ONFH Susceptibility

Because LD is believed to be highly structured, with conserved blocks of sequence separated by hotspots of recombination, the function of a conserved haplotype may result from interaction between polymorphisms within a block. Therefore, SNP haplotypes were then constructed on the basis of genotypes of the SNPs, which resided in LD block (Figure 1(b)). Four major haplotypes with frequencies > 0.05 were predicted in LD block 1, and the frequency of each haplotype was compared between SLE and SLE_ONFH patients (Tables 4 and 5). Haplotype 3 (ht3: T-G-T-G-A) and haplotype 4 (ht4: C-C-T-A-A) were associated with an increased risk (OR; 3.73~) of ONFH in SLE patients under recessive analysis model (Table 4). None of haplotypes located in block 2 showed evidence for association (data not shown). These results suggest that polymorphisms located in extracellular domain of* CR2* gene may be functionally involved with increased susceptibility to ONFH in SLE patients.

## 4. Discussion

Although ONFH is a common complication deteriorating the treatment of SLE, details of the pathogenesis are not well established. Because venous thrombosis and resultant blood flow obstruction mediated by thrombophilia or hypofibrinolysis are generally assumed to develop ONFH [18, 19], most of gene studies have focused on gene polymorphisms affecting the coagulation and fibrinolytic systems [8, 9]. Recent studies, however, reported that immunologic factors might develop ONFH [6, 7] and few genetic studies were performed to reveal their roles in the development of SLE_ONFH [10].

Complement receptor type 2 (*CR2*) is a membrane glycoprotein that binds C3 degradation products generated during complement activation, specifically iC3b, C3dg, and C3d. It has many important functions in normal immunity, such as targeting antigen to follicular dendritic cells in secondary lymphoid organs and cooperating with the B cell receptor to activate B cells [13].* CR2* is also assumed to play a role in the development of autoimmune disease [16]. Therefore, given these pleiotropic effects of complement receptor, we investigated whether polymorphisms of the* CR2* gene were associated with the development of SLE_ONFH.

In this study, the SNPs rs3813946 in 5′-UTR, rs311306 in intron 1, and rs17615 in exon 10 (nonsynonymous SNP; G/A, Ser639Asn) of the* CR2* gene are associated with the susceptibility of SLE_ONFH under recessive model. Haplotype T-G-T-G-A (ht3) and haplotype C-C-T-A-A (ht4) (SNP order of haplotypes: rs3813946-rs311306-rs1567190-rs17615-rs17045328) were also associated with an increased risk (OR; 3.73~) of SLE_ONFH (Table 4). However, when Bonferroni correction for multiple testing was applied, there was no significance in all SNPs and haplotypes. Previously, it was reported that the minor C allele of rs3813946, located in 5′UTR of* CR2*, reduced transcription of reporter genes in CR2-nonexpressing erythroleukemia cells [13] and CR2 expressing B cells [16]. Under basic conditions, primary B cells from individuals homozygous or heterozygous for the minor allele at rs3813946 demonstrated a trend toward reduced levels of CR2 RNA transcript [16]. Nonsynonymous SNP rs17615, which is located in exon 10 of the* CR2* gene, might also have functional effects that could lead to disease. Exon 10 is located directly in 5′ of alternatively spliced exon 10a, which is found in a long CR2 isoform [20]. SNPs in coding region can change pre-mRNA splicing and message stability [21], and rs17615 allelic variant may regulate the relative level of the long and short isoform of* CR2 *[16]. Alternative splicing can be involved in the process of regulating normal physiological functions as well as pathologies. Genomewide alternative splicing studies estimate that greater than 95% of human multiexon genes express multiple splice isoforms. Interindividual variation in isoforms resulting from SNPs located in splicing regulatory motifs can occur in up to 21% of alternatively spliced genes [22], and the effects of these on splicing efficiency are assumed to contribute significantly to disease severity as well as susceptibility [23]. Alterations of* CR2* expression have variable different effects on manifestations of disease in animal models of autoimmunity [24]. In addition, the substitution of asparagine for serine (rs17615; Ser639Asn), which is conserved in mice, rats, and sheep, may be important in receptor function. Therefore, there is a possibility that the dysregulation expression of* CR2* is associated with the occurrence of the SLE_ONFH. Although the current study showed positive relationship between* CR2* polymorphisms and SLE_ONFH, it is also limited. First, we had limited basic and clinical data of study samples. Important information such as family history of ONFH, onset of diseases, and medication history was not available in this study. Second, our study sample size is not enough for analysis of the effect of* CR2* gene in ONFH with SLE. Although ONFH is one of the most common diseases around the hip joint in Korea, the incidence is relatively low in most countries. According to medical claims data from Korean National Health Insurance Corporation, the estimated average number of annual prevalent cases was 28.9 per 100,000. Moreover, the incidence of ONFH in SLE patients is very low. We did not get enough samples for obtaining high power in the association analysis. Nevertheless, we believe that our findings are valuable because this is first study to reveal the association between polymorphisms of the* CR2* gene and susceptibility of SLE_ONFH. This study will promote the replication study of other researchers on the suggestive results and thus will improve our understanding of ONFH pathogenesis in SLE patients.

In conclusion, three* CR2* polymorphisms are associated with ONFH susceptibility in SLE patients in our case-control study and these findings may provide evidence that* CR2* contributes to human ONFH susceptibility in SLE patients. However, further well-designed studies with large sample size are mandatory for establishing our findings and reveal clinical importance of them.

## Figures and Tables

**Table 1:** List of TaqMan probes for SNP genotyping of *CR2* SNPs.

TaqMan genotyping SNP ID	Probes (ABI)	Context sequence [VIC/FAM]
rs3813946	C_25599654_10	CTCACAGCTGCTTGCTGCTCCAGCC**[C/T]**TGCCCTCCCAGAGCTGCCGGACGCT
rs311306	C_1009912_10	ACCTATCACCATCTAAAACCTTCTT**[C/G]**CTTATTTATGTACCTGTTTGTTGTT
rs1567190	C_8827000_20	GGAGTATAGGCTACATAGTGAGGAA**[C/T]**GGTAGTTGATTAAATGGATTGGAGC
rs17615	C_12082973_1_	TATAGTGGATTTACTTTGAAGGGCA**[A/G]**TAGTCAGATTCGTTGCAAAGCTGAT
rs17045328	C_32843029_10	ATTCTAACCTGAGAAATCTCTGATT**[A/G]**TAAAGTTGAGTATATTGTTTTTGTC
rs12032512	C_26228419_10	AACCTTAAGCTCAACTATGATTATT**[C/G]**AGGAATTCAGCATTTATGTCCAAGA
rs6690215	C_30168452_10	TTGGTGAGGATGCAAAGCAAATGGT**[C/T]**AATATTTGGGAGTTTTAATCAGGAA
rs17045468	C_32842997_10	GGGTCTCAAAAAAAATTAGGGATGT**[C/T]**ATTTGCAGGGCCTTCCTCATAGGAA

VIC: 2′-chloro-7′-phenyl-1,4-dichloro-6-carboxyfluorescein. VIC is a fluorescent dye that was originally developed by Applied Biosystems.

FAM: 6-carboxyfluorescein.

**Table 2:** SNP markers in the *CR2* gene genotyped in this case-control study.

rs number (alternative name)	Position	Amino acid substitution	Genotype			MAF^(1)^		HWE^(2)^
			C/C^(3)^	C/R	R/R	Control	Case	
rs3813946 (T/C)	*Exon (5*′* UTR)*	No	TT	CT	CC	0.144	0.184	0.309
rs311306 (G/C)	*Intron 1*	No	GG	CG	CC	0.139	0.20	0.299
rs1567190 (T/C)	*Intron 1*	No	TT	CT	CC	0.419	0.34	0.864
rs17615 (G/A)	*Coding exon 10*	Ser639Asn (A**G**T-A**A**T)	GG	AG	AA	0.133	0.163	0.077
rs17045328 (A/G)	*Intron 16*	No	AA	AG	GG	0.262	0.208	0.517
rs12032512 (C/G)	*Intron 17*	No	CC	CG	GG	0.419	0.45	0.396
rs6690215 (C/T)	*Intron 18*	No	CC	CT	TT	0.463	0.43	0.618
rs17045468 (C/T)	*Intron 18*	No	CC	CT	TT	0.255	0.23	0.379

^(1)^MAF: minor allele frequency; ^(2)^HWE: *P* values of deviation from Hardy-Weinberg equilibrium.

^(3)^C/C: major homozygote; C/R: heterozygote; R/R: minor homozygote.

**Table 3:** Analyses of association between *CR2* gene polymorphisms and the risk of ONFH in SLE patients.

SNP rs number	Genotype	Frequencies (%)		Allele		Dominant		Recessive	
		Controls (SLE)	Cases (SLE_ONFH)	OR (95% CI)	*P*	OR (95% CI)	*P*	OR (95% CI)	*P*
rs3813946	TT	102 (71.83)	35 (71.43)						
	CT	39 (27.46)	10 (20.41)	1.33 (0.73–2.45)	0.353	1.02 (0.50–2.10)	0.957	**12.5 (1.37–115)**	0.016^*∗*^
	CC	1 (0.7)	4 (8.16)						

rs311306	GG	108 (72.97)	35 (70.0)						
	CG	39 (26.35)	10 (20.0)	1.56 (0.86–2.81)	0.141	1.16 (0.57–2.34)	0.685	**16.3 (1.86–143)**	0.004^*∗*^
	CC	1 (0.68)	5 (10.0)						

rs1567190	TT	49 (33.11)	22 (44.0)						
	CT	74 (50.0)	22 (44.0)	0.72 (0.45–1.15)	0.164	0.63 (0.33–1.21)	0.165	0.67 (0.26–1.74)	0.411
	CC	25 (16.89)	6 (12.0)						

rs17615 (S639N)	GG	108 (73.47)	36 (73.47)						
	AG	39 (26.53)	10 (20.41)	1.28 (0.68–2.40)	0.450	1.0 (0.48–2.08)	1		0.015^*∗*^
	AA	0 (0)	3 (6.12)						

rs17045328	AA	78 (53.06)	31 (64.58)						
	AG	61 (41.5)	14 (29.17)	0.74 (0.43–1.29)	0.292	0.62 (0.32–1.22)	0.163	1.16 (0.30–4.55)	0.734^*∗*^
	GG	8 (5.44)	3 (6.25)						

rs12032512	CC	47 (31.76)	16 (32.0)						
	CG	78 (52.7)	23 (46.0)	1.14 (0.72–1.79)	0.587	0.99 (0.50–1.97)	0.975	1.53 (0.69–3.42)	0.295
	GG	23 (15.54)	11 (22.0)						

rs6690215	CC	41 (27.7)	20 (40.0)						
	CT	77 (52.03)	17 (34.0)	0.88 (0.55–1.38)	0.569	0.58 (0.29–1.12)	0.103	1.38 (0.65–2.92)	0.396
	TT	30 (20.27)	13 (26.0)						

rs17045468	CC	79 (53.74)	30 (60.0)						
	CT	61 (41.5)	17 (34.0)	0.87 (0.51–1.49)	0.616	0.78 (0.40–1.49)	0.442	1.28 (0.32–5.14)	0.716^*∗*^
	TT	7 (4.76)	3 (6.0)						

Genotype distributions are shown as number (%). Chi-square *P* values and odds ratio (95% CI) are shown. OR: odds ratio; CI: confidence interval.

^*∗*^Fisher's exact test.

**Table 4:** Analyses of association between *CR2* gene haplotypes and the risk of ONFH in SLE patients.

Haplotype	Genotype	Frequencies (%)		Allele		Dominant		Recessive	
		SLE	SLE_ONFH	OR (95% CI)	*P*	OR (95% CI)	*P*	OR (95% CI)	*P*
Block1-ht1 T-G-C-G-A	*−/−*	46 (32.86)	22 (45.83)						
	*ht1/−*	71 (50.71)	21 (43.75)	0.66 (0.41–1.08)	0.10	0.58 (0.30–1.13)	0.106	0.59 (0.21–1.65)	0.313
	*ht1/ht1*	23 (16.43)	5 (10.42)						

Block1-ht2 T-G-T-G-G	*−/−*	74 (52.86)	31 (64.58)						
	*ht2/−*	58 (41.43)	14 (29.17)	0.73 (0.42–1.28)	0.275	0.62 (0.31–1.21)	0.158	1.1 (0.28–4.33)	1.0
	*ht2/ht2*	8 (5.71)	3 (6.25)						

Block1-ht3 T-G-T-G-A	*−/−*	96 (68.57)	28 (58.33)						
	*ht3/−*	38 (27.14)	13 (27.08)	1.8 (1.05–3.09)	0.031	1.56 (0.79–3.06)	0.196	**3.81 (1.21–12.0)**	0.023^*∗*^
	*ht3/ht3*	6 (4.29)	7 (14.58)						

Block1-ht4 C-C-T-A-A	*−/−*	101 (72.14)	35 (72.92)						
	*ht4/−*	39 (27.86)	10 (20.83)	1.24 (0.66–2.33)	0.512	0.96 (0.46–2.01)	0.918		0.016^*∗*^
	*ht4/ht4*	0 (0.0)	3 (6.25)						

(i) SNP order of haplotypes: rs3813946 (T/C)-rs311306 (G/C)-rs1567190 (T/C)-rs17615 (G/A)-rs17045328 (A/G).

(ii) Haplotype distributions are shown as number (%). Chi-square *P* values and odds ratio (95% CI) are shown. OR: odds ratio; CI: confidence interval.

(iii) ^*∗*^Fisher's exact test.

**Table 5:** Haplotypes of CR2 in blocks 1 and 2 shown in Figure 1.

Haplotype ID	Haplotypes		Frequencies
Block 1	*ht1*	TGCGA	0.407
	*ht2*	TGTGG	0.255
	*ht3*	TGTGA	0.196
	*ht4*	CCTAA	0.142

Block 2	*ht1*	TC	0.449
	*ht2*	CC	0.287
	*ht3*	CT	0.262
